# Tumor lysis syndrome in chronic lymphocytic leukemia: conventional treatment versus novel agents: A protocol for systematic review and meta-analysis

**DOI:** 10.1097/MD.0000000000023632

**Published:** 2020-12-18

**Authors:** Nancy Kassem, Ahmed A. Ghazy, Mohammad Abu-Tineh, Nabil E. Omar, Abdulqadir J. Nashwan, Prem Chandra, Rola Ghasoub, Osama S. AbuTabar, Mohamed A. Yassin

**Affiliations:** a Pharmacy Department, National Center for Cancer Care and Research; b Bariatric Medicine Department; c Medical Oncology/Hematology Department, National Centre for Cancer Care and Research; d Nursing Department, Hazm Mebaireek General Hospital; e Medical Research Center Hamad Medical Corporation, Doha, Qatar; f Pharmacy Department, Cleveland Clinic Abu Dhabi & MSc Experimental and Translational Therapeutics Candidate, University of Oxford-Kellogg College.

**Keywords:** chronic lymphocytic leukaemia, conventional chemotherapeutic agents, novel chemotherapeutic agents, tumor lysis syndrome

## Abstract

**Introduction:**

Existing evidence on the difference in the incidence of tumor lysis syndrome (TLS) in Chronic Lymphocytic Leukemia (CLL) patients receiving novel therapies versus patients receiving conventional therapies is limited and inconclusive. The aims of this planned systematic review and meta-analysis are therefore

(1) assess the TLS incidence reported in clinical trials for the novel or targeted agents comparing to conventional chemotherapeutic agents used to treat patients with CLL
(2) to identify the TLS prophylaxis strategies that are utilized in clinical trials of the novel or targeted agents for CLL if it was fully reported or under-reported and
(3) to compare the mortality among patients with TLS in conventional versus novel agents.

**Methods:**

We will conduct a systematic review and meta-analysis. Several electronic databases will be searched using predefined search terms to identify relevant studies. Eligible studies should report findings on the incidence of TLS in CLL patients. Primary observational studies with cross-sectional or prospective research design, case-control studies, and studies with experimental designs will be included. Study quality will be evaluated by 2 reviewers using the statistical methodology and categories described in the Cochrane Collaboration Handbook and preferred reporting items for systematic reviews and meta-analyses and other applicable guidelines. The meta-analysis will be performed and conducted using applicable standard statistical software like comprehensive meta-analysis and STATA.

**Discussion:**

This review and meta-analysis will be among the first to systematically explore and integrate the evidence available on the comparison between the incidences of TLS in CLL patients treated with novel agents versus conventional agents. By gathering and summarizing information about the risk of TLS in this patient population, the findings from this review will provide insights for future research directions and more understanding of the difference of TLS incidence between novel treatments and conventional treatment and suggest prophylactic measures for such cases.

**Systematic review registration:**

The protocol has been registered at the International Prospective Register of Systematic Reviews (PROSPERO; registration number: CRD42020166770).

The protocol was registered with the Hamad medical corporation, Medical research Center registry under a unique reference number (MRC-01-20-709).

## 1 Introduction

Chronic lymphocytic leukemia (CLL) is 1 of the chronic lymphoproliferative disorders (lymphoid neoplasms). It is characterized by a progressive accumulation of functionally incompetent lymphocytes, which are usually monoclonal in origin.^[1]^ Tumor lysis syndrome (TLS) is an oncologic emergency that is caused by massive tumor cell lysis with the release of large amounts of potassium, phosphate, and nucleic acids into the systemic circulation.^[2]^ Laboratory TLS was defined as any 2 or more of the following: hyperkalemia, hyperphosphatemia, hyperuricemia, or hypocalcemia, present within 3 days before or 7 days after instituting chemotherapy.

Clinical TLS was defined as laboratory TLS plus 1 or more of the following that was not directly or probably attributable to a therapeutic agent: increased serum creatinine concentration (≥1.5 times the ULN), cardiac arrhythmia/sudden death, or a seizure.^[3]^

The incidence and severity of TLS depend on the type of disease and the type of treatment. Most often, it occurs after the initiation of cytotoxic therapy in patients with high-grade lymphomas (particularly the Burkitt's leukemia subtype) and acute lymphoblastic leukemia. However, it can occur spontaneously and with other tumor types that have a high proliferative rate, large tumor burden, or high sensitivity to cytotoxic therapy.^[4,5]^

Multiple effective treatments are available for patients with CLL. However, the risk of TLS associated with treatment hasn’t been systemically examined or analyzed. A literature review identified multiple phases I to III clinical trials of Monoclonal antibodies, tyrosine kinase inhibitors, proteasome inhibitors, chimeric antigen receptor T cell therapy, and pro-apoptotic agents like lenalidomide.^[6–16]^

This planned study will systematically review all available literature on the incidence of TLS in patients with CLL treated with novel versus conventional therapies.

## 2 Methods

The following protocol has been written according to the meta-analyses of observational studies in epidemiology Guidelines for Meta-Analyses and Systematic Reviews of Observational Studies and the PRISMA-P (preferred reporting items for systematic reviews and meta-analyses) guidelines.^[17,18]^ The protocol has been registered at the International Prospective Register of Systematic Reviews (PROSPERO; registration number: CRD42020166770).

### 2.1 Data sources search terms and search strategy

To achieve the study objectives, searches will be carried out in the following electronic databases: PubMed, Scopus, Web of Science, Web of Conferences, Open Grey. The following search terms will be used in literature review: (“chronic lymphocytic leukemia” OR “well-differentiated lymphocytic lymphoma” OR “small lymphocytic lymphoma” OR “small-cell lymphoma” OR “chronic Lymphoblastic Leukemia” OR “Lymphocytic Lymphoma” OR “Chronic B-Cell Leukemia” OR “Chronic B-Lymphocytic Leukemia” OR “CLL” OR “low-grade lymphoma”) AND (“tumor lysis syndrome” OR “tumor lysis syndrome” OR “TLS”

Reference lists of key full-text and any reports that may be eligible will be reviewed for publications included in the study. The systematic method specifies that all published research constitute the literature search on CLL and TLS. The search strategy is considered adequate to reduce the risk of selection and detection bias. The search results will be exported to Endnote where duplicates are excluded. Included studies will be manually screened to select other relevant studies.

A populated PRISMA-P checklist was used as an aid to authors to clearly, completely, and transparently let reviewers and readers know what authors intend to do.^[19]^

### 2.2 Inclusion and exclusion criteria

#### 2.2.1 Types of studies

Eligible studies should report the empirical incidence of TLS in CLL patients treated with novel and/or conventional therapies. All literature including clinical trials, case reports, case series, and abstracts from 2009 to 2020 in the English language will be included. Data based on conference abstracts and gray literature (eg, reports, etc) will also be included. Studies in languages other than English will not be included.

#### 2.2.2 Participants

The study population will include adult (18 years or older) patients diagnosed with CLL diagnosis and received treatment. Exclusion criteria include patients aged less than 19 years, known active histological transformation from CLL to an aggressive lymphoma (ie, Richter transformation), other diagnoses of active cancer or the presence of other active malignancy or the use of systemic therapy for another malignancy within 3 years; local/regional therapy with curative intent years of treatment is permitted.

### 2.3 Data extraction (selection and coding)

Two reviewers will independently assess eligibility of all the citations described and extracted data from the original trial reports using a limited data extraction method including the study details for example publication year, authors, study design, clinicalTrials.gov Identifier code, follow-up duration), patient characteristics (inclusion and exclusion criteria and other various related features), sample size and the details of interventions/comparisons, primary and secondary outcome measures, and subgroup/stratified statistical analyses reported included.

Statistical evaluation of both safety and efficacy parameters using various statistical measures such as hazard ratios (HRs), progression-free survival (PFS), and overall survival (OS) between different treatments will be reported.

To minimize any data entry error, all data will be entered in duplicate and cross-checked for accuracy, and disparities would be discussed in a team meeting. Assessment of study quality or strength of study will be carried out as well (high, moderate, low)

### 2.4 Assessment of methodological quality (risk of bias)

Study quality will be evaluated by 2 reviewers using the statistical methodology and categories described in the Cochrane Collaboration Handbook and PRISMA and other applicable guidelines. In case of disagreement, a team meeting/group discussion will be conducted to reach a consensus. Other potential issues will also be considered that includes baseline imbalance and the other potential issues. The cumulative evidence might be affected by bias (eg, publication bias, selective reporting within studies) will be evaluated. Risk of bias that might affect the cumulative evidence (such as publication bias, selective reporting within studies) will be assessed with plotting the effect by the inverse of its standard error. The symmetry of such ‘funnel plots’ (using applicable standard statistical software like comprehensive meta-analysis and STATA etc) will be assessed both visually, and formally with Egger test, to see if the effect decreased with increasing sample size.

### 2.5 Meta-analytic approach

The meta-analysis will be performed and conducted using applicable standard statistical software like comprehensive meta-analysis and STATA and so on. Confidence intervals will be set at 95%. The inter-study heterogeneity will be evaluated with the inconsistency index (*I*^2^). When significant heterogeneity is present (*I*^2^ > 50%), a random-effects model will be implemented to calculate pooled estimates of specific effect size measures along with the 95% confidence intervals (95% CI).

Subgroup analysis will be conducted depending on potential factors and covariates that might affect mainly primary outcome measures. Sensitivity analysis will be performed to investigate the effect of individual studies concerning the primary outcome measures of the meta-analysis.

A network meta-analysis will be conducted to compare the treatment outcomes between conventional treatments and several novel targeted agents, and all results will be reported according to the PRISMA extension statement for network meta-analyses.

A network meta-analysis combines direct and indirect estimates of relative treatment effects in 1 statistical analysis.

A network plot will be produced to represent the data from all trials included in the analysis. The contribution of each direct comparison to the network estimate will be calculated according to the variance of the direct treatment effect and the network structure, later summarized in a contribution plot.

A forest plot of the estimated summary effects, along with CIs for all comparisons, summarizes the relative mean effect or other effect size measure and prediction on each comparison in 1 plot.

Statistical analysis based on potential subgroups and stratifications (eg, stage and severity of the disease, age group, gender, clinical significance, and co-morbidity etc) that might affect primary outcome measures.

## 3 Discussion

This systematic review and meta-analysis study will estimate the pooled incidence of TLS in patients with CLL with novel and conventional therapies. It will also provide information about the prophylactic measures used. Since this study will use comprehensive and meticulous methods in all the steps of the systematic review and meta-analysis, the information obtained will be completely reliable.

## 4 Limitations

Methodological biases in the primary studies included may cause uncertainty in the results of the present study.

## 5 Ethics and dissemination

Ethical approval is not required for this systematic review and meta-analysis as only a secondary analysis of data already available in scientific databases will be conducted. The results of this review will be submitted for peer-reviewed publication and will be presented at relevant conferences.

The protocol was registered with the Hamad medical corporation, Medical research Center registry under a unique reference number (MRC-01-20-709)

## Author contributions

NK, AG, MAA, NEO, AJN, PC, RG, OSA, MAY: Data Collection, Literature Search, Manuscript Preparation.

All authors read and approved the final manuscript

**Conceptualization:** Nancy A. Kassem, Ahmed A. Ghazy, Mohammad Abu-Tineh, Nabil Elhadi Omar, Abdulqadir J. Nashwan, Rola Ghasoub, Osama S. AbuTabar, Mohamed A. Yassin.

**Data curation:** Nancy A. Kassem, Ahmed A. Ghazy, Mohammad Abu-Tineh, Nabil Elhadi Omar, Abdulqadir J. Nashwan, Rola Ghasoub, Osama S. AbuTabar, Mohamed A. Yassin.

**Formal analysis:** Prem Chandra.

**Methodology:** Ahmed A. Ghazy, Prem Chandra.

**Project administration:** Osama S. AbuTabar, Mohamed A. Yassin.

**Software:** Prem Chandra.

**Supervision:** Mohamed A. Yassin.

**Validation:** Prem Chandra.

**Visualization:** Prem Chandra.

**Writing – original draft:** Nancy A. Kassem, Ahmed A. Ghazy, Mohammad Abu-Tineh, Nabil Elhadi Omar, Abdulqadir J. Nashwan, Rola Ghasoub, Osama S. AbuTabar, Mohamed A. Yassin.

**Writing – review & editing:** Nancy A. Kassem, Mohammad Abu-Tineh, Abdulqadir J. Nashwan, Prem Chandra, Rola Ghasoub, Osama S. AbuTabar, Mohamed A. Yassin.
